# Local Treatment of Breast Cancer Liver Metastasis

**DOI:** 10.3390/cancers11091341

**Published:** 2019-09-11

**Authors:** Reto Bale, Daniel Putzer, Peter Schullian

**Affiliations:** Department of Radiology, Section of Interventional Oncology-Microinvasive Therapy, Medical University of Innsbruck, 6020 Innsbruck, Austria; daniel.putzer@tirol-kliniken.at

**Keywords:** breast cancer, liver metastasis, local recurrence, survival, metastasectomy, resection, thermal ablation, stereotactic radiofrequency ablation (SRFA), stereotaxy, image fusion

## Abstract

Breast cancer represents a leading cause of death worldwide. Despite the advances in systemic therapies, the prognosis for patients with breast cancer liver metastasis (BCLM) remains poor. Especially in case of failure or cessation of systemic treatments, surgical resection for BCLMs has been considered as the treatment standard despite a lack of robust evidence of benefit. However, due to the extent and location of disease and physical condition, the number of patients with BCLM who are eligible for surgery is limited. Palliative locoregional treatments of liver metastases (LM) include transarterial embolization (TAE), transarterial chemoembolization (TACE), and selective internal radiotherapy (SIRT). Percutaneous thermal ablation methods, such as radiofrequency ablation (RFA) and microwave ablation (MWA), are considered potentially curative local treatment options. They are less invasive, less expensive and have fewer contraindications and complication rates than surgery. Because conventional ultrasound- and computed tomography-guided single-probe thermal ablation is limited by tumor size, multi-probe stereotactic radiofrequency ablation (SRFA) with intraoperative image fusion for immediate, reliable judgment has been developed in order to treat large and multiple tumors within one session. This review focuses on the different minimally invasive local and locoregional treatment options for BCLM and attempts to describe their current and future role in the multidisciplinary treatment setting.

## 1. Introduction

Breast cancer is a leading cause of mortality worldwide [1]. It is a heterogeneous disease with specific molecular subtypes, which are associated with different prognosis and response to treatment. Approximately 50% of all women diagnosed with breast cancer develop metastatic disease. The common metastatic sites are liver, lung, bone, and brain. Liver metastases develop in approximately 50% of all patients with metastatic breast cancer and 5–12% of patients develop liver metastases as the primary site of breast cancer recurrence [2].

Metastatic liver disease may cause impairment of liver function and endanger the patients’ life. If left untreated, liver metastases (LM) are associated with poor survival ranging from 4 to 8 months [3]. Patients with advanced disease are primarily treated by systemic hormone- and/or chemotherapy [4,5]. However, despite an improvement in systemic treatment, median survival of patients with metastatic disease from the time of diagnosis of metastatic breast carcinoma is approximately 18–24 months, and 5-year and 10-year survival rates are still as low as 27% and 13%, respectively [6]. Despite transient response to chemotherapy or endocrine therapy, most patients exhibit progressive disease changes after 1–2 years [7].

Recent studies demonstrated that subgroups of these heterogeneous patients with oligometastatic disease benefit from additional local or locoregional treatment, with improved survival rates after R0 resection of breast cancer liver metastasis (BCLM) when compared to systemic treatment alone [8,9,10]. According to the 3rd ESO–ESMO (European School of Oncology–European Society for Medical Oncology) International Consensus Guidelines for Advanced Breast Cancer 3 (ABC 3) oligometastatic disease is defined as low volume metastatic disease with limited number and size of metastatic lesions (up to five and not necessarily in the same organ) that are potentially amenable for local treatment to achieve long-term remission [11]. The ideal local treatment would be minimally invasive with a low morbidity and mortality rate. This review tries to identify the current and potential future role of local treatment of BCLM in general and focuses on the application of minimally invasive interventional oncologic interventions in the multimodal treatment setting of BCLM.

## 2. Materials and Methods

### Search

A literature search was performed using the Medline/PubMed database to identify studies reporting on locoregional treatment or metastasectomy for patients with BLCM. Search terms used were (“breast cancer”) AND (“liver metastases”) OR “hepatic metastases” OR “liver metastasis” OR “hepatic metastasis”) AND (metastasectomy OR “hepatic resection” OR hepatectomy OR ablation OR radiotherapy OR radioembolization OR “transarterial embolization” OR “stereotactic radiofrequency ablation”) AND “survival”.

In addition, all “similar articles” as listed in the right column on the PubMed homepage were reviewed. Only studies in the English language, published between 2000 and June 2019 with inclusion of a minimum of ten patients were considered. Publications in other languages, case reports, preclinical studies, or reviews were excluded. A manual search of references of retrieved articles for additional relevant publications was performed. After removal of duplicates, then screening by abstract, title, and full text, selected studies that met the inclusion criteria were subsequently reviewed. Consensus was required for inclusion by two authors (B.R., P.S.).

## 3. Results

We identified a total of 201 studies. Among them, 157 were excluded (25 papers published before 2000, 26 reviews, seven case reports, and nine publications in other languages, 23 publications dealing with locoregional treatment of liver metastases from other origins, seven publications reporting technical issues only, 60 publications dealing with focus on systemic treatment or insufficient survival data). Eventually, a total of 44 studies reporting on surgery (*n* = 24), radiofrequency ablation (*n* = 9), kryoablation (*n* = 1), radioembolization (*n* = 5), transarterial chemoembolization (*n* = 2), brachytherapy (*n* = 2) and stereotactic body radiation therapy (SBRT) (*n* = 4) in BCLM patients were considered. The studies are discussed in the relevant sections below and summarized in Table 1 and Table 2.

### 3.1. Resection of BCLM 

In contrast to the substantial evidence for local treatment of colorectal liver metastases, the data for resection of BCLM are limited. In heterogenous case series the reported median 3-, and 5-year survival rates after metastasectomy of BCLM range between 24–116 months, and 49–94% and 5–78%, respectively [3,10,21,22,23,24,25,26,27,28,29,30,31,32,33,34,35,36,37,38,39,40,41,42]. In a systematic review Fairhurst et al. [55] analyzed 33 papers dealing with resection of BCLM in a total of 956 patients. The mortality ranged between 0% and 5.9% and the median morbidity rate was 15%. The median overall survival (OS) was 35.1 months, with a median 1-, 2-, 3-, and 5-year survival of 84.6%, 71.4%, 52.9%, and 33% respectively. The median disease-free survival (DFS) was 21.5 months with a 3- and 5-year median DFS of 36% and 18%. In a more recent paper, Ercolani et al. reported a 10-year OS rate of 16% in an updated single center experience in 51 patients [27]. In a case-matched analysis, patients from the Netherlands with BCLM who received systemic treatment only were compared with patients from France who received a combination of systemic treatment with hepatectomy. After matching, the resection group had a median OS of 82 months with a 3- and 5-year OS of 81% and 69%, respectively, compared with a median OS of 31 months in the systemic group with a 3- and 5-year OS of 32% and 24%, respectively [10]. The authors concluded that for patients with BCLM, liver resection combined with systemic treatment results in improved OS compared to systemic treatment alone. 

The major drawback of most studies is the poor data quality due to the inclusion of small numbers of patients and multiple confounding variables including tumor biology of the primary tumors, presence of synchronous or metachronous extrahepatic metastases, systemic treatments and time intervals between primary tumor and systemic treatment and the type of local treatment of BCLM. Most patient cohorts in the surgical series are highly selected and it remains unclear whether the resection itself or the favorable tumor biology is responsible for the results.

Despite some promising reports, surgical resection of BCLM is still controversial because of its invasiveness. In addition, many patients develop unpredictable recurrent disease [56]. Liver recurrences and extrahepatic recurrences were diagnosed at a mean interval of 15 months and 22 months after hepatectomy [57].

#### 3.1.1. Prognostic Factors

In order to select the proper patients, it is crucial to find out independent factors that influence the prognosis after BCLM resection. Characteristics of primary breast cancer such as small tumor size, low grade, node negativity, and early stage may be associated with better outcome after liver metastasectomy [39,40,41,58]. Moreover, response to preoperative systemic therapy has been identified as a prognostic factor which is likely related to effective systemic eradication of microscopic metastatic lesions [3,21,30]. In addition, complete macroscopic and microscopic resection (R0) [3,29,30,32,40], liver-limited disease (with the exception of isolated pulmonary and bony metastases) [23,39,59], solitary BCLM [36,41], a long interval (more than 1 year) between breast cancer diagnosis and the detection of BCLM [21,28,29,35,37,57] and patients with PgR- and/or ER-positive BCLM [21,22,23,27,30,34,60] were independent prognostic factors. In conclusion at least a selected group of patients with BCLM benefits from aggressive local curative treatment. Further studies are required to define more specific selection criteria for local treatment of BCLM.

### 3.2. Non-Surgical Local Treatment Options with Palliative Intent

For various reasons the majority of patients are unresectable at the time of diagnosis of BCLM [10,61]. In addition, alternative minimally invasive treatment options that achieve equal local control but with lower morbidity and mortality as compared to surgical resection would be highly desirable.

Transarterial locoregional therapies including transcatheter arterial embolization (TAE), transcatheter arterial chemoembolization (TACE) [53,62,63], and selective internal radiation therapy (SIRT) [46,47,48,49,50,51,52] have been introduced with the primary goal of palliation. They are based on the observation from animal studies that hepatic tumors are mainly supplied from the hepatic artery as opposed to the portal vein. They have been developed to deliver high doses of chemotherapeutic (TACE) or radioactive agents (SIRT) directly to the target tumor and to prolong drug/radiation exposure to the tumor cells while minimizing systemic side effects.

#### 3.2.1. Transarterial Chemoembolization (TACE)

In TACE [64] high doses of chemotherapeutic agents are directly delivered to the target tumor. In addition, the chemotherapeutic effect of TACE on tumor cells is augmented by the embolization induced ischemia. It is well established for the palliative treatment of hepatocellular carcinoma [65]. TACE is a minimally invasive procedure associated with a very short hospital stay and minimal side effects. However, there is only sparse data available on the application of TACE for patients with BCLM.

Li et al. [54] reported the results of TACE and systemic chemotherapy for 46 patients with BCLM. After a median follow-up of 28 months response rates for the TACE group and chemotherapy group, were 35.7% and 7.1%, respectively. The 1-, 2-, and 3-year respective survival rates for the TACE group were 63.0%, 30.4%, and 13.0%, and those for the systemic chemotherapy group were 33.9%, 11.3%, and 0%. 

The role of TACE in unresectable BCLM was also evaluated by Cho et al. [62] in a retrospective review of ten patients treated by a median number of four TACE sessions. An increase in median survival was observed for patients who responded to treatment when compared to non- responders (24 vs. 7 months, *p* = 0.02). In a prospective phase II study, Eichler et al. [53] evaluated the efficacy and tolerability of TACE with gemcitabine in 43 patients with inoperable BCLM. All patients tolerated the treatment well. Follow-up imaging revealed a partial response in three patients, stable disease in 16 patients, and progression in 22 patients, resulting in a progression-free survival of 3.3 months, and an estimated median survival rate of 10.2 months. 

#### 3.2.2. Selective Internal Radiation Therapy (SIRT)

SIRT was originally developed as a liver-directed therapy for primary liver cancer and colorectal liver metastases. A randomized multicenter clinical trial showed an improvement in radiological response rate and hepatic progression-free survival in patients with colorectal liver metastases treated with SIRT [66]. SIRT is based on the administration of yttrium-90 (90 Y) microspheres with a diameter of approximately 30 μm via the arterial blood supply of liver tumors. Adverse events include radioembolization-induced liver disease (REILD), postradioembolization syndrome (PRS), biliary complications, radiation pneumonitis, gastroduodenal ulceration, lymphopenia, vascular injury, and portal hypertension [67]. REILD is characterized by jaundice and ascites 1 to 2 months after SIRT without bile duct occlusion or tumor progression, which occurs in up to 20% of cases and seems to be associated with the combined effect of radiation and chemotherapy [68]. 

PRS typically consists of unspecific symptoms including fatigue, anorexia, and fever and is commonly observed after SIRT but is mild, requiring only symptomatic management. Several retrospective studies have explored the use of SIRT in patients with BCLM refractory to systemic treatment with a median OS of 4–13.6 months [46,47,48,49,50,51,52]. Haug et al. reported a median OS of 11.8 months in 58 women receiving SIRT for BCLM [49]. Gordon et al. achieved a partial response of 35.3% and stable disease in 63.2%, with a median OS of 6.6 months in 75 patients [48]. One systematic review included 198 patients from six retrospective cohort studies. Disease control (complete response, partial response or stable disease) was observed in 78–96% at 2–4 months [69]. The absence of extrahepatic disease [50,51], response to SIRT [49,51,52] and a low liver tumor burden have been associated with good prognosis [48,51,52].

### 3.3. Non-Surgical Local Treatment Options with Curative Intent

#### 3.3.1. Stereotactic Body Radiation Therapy (SBRT)

The liver parenchyma has low radiation tolerance doses. However, by delivering higher doses to small volumes, organ function can be maintained without causing functional compromise [67]. Due to the delivery of conformal doses and steep dose gradients SBRT allows normal liver tissues to be spared. Retrospective and prospective studies have demonstrated the feasibility of SBRT for LM from different tumor entities with local control (LC) rates ranging from 60–90% at 2 years after treatment [70,71]. In a recent paper, Onal et al. [43] combined liver SBRT and systemic treatment in a total of 22 patients with 29 BCLM, with a mean size of 2.1 ± 1.2 cm. After a median follow-up time of 16.0 months (range 4.4–59.4 months), 18 patients (82%) had disease recurrence. The 1- and 2-year OS rates were 85% and 57%, and the 1- and 2- year PFS rates were 38% and 8%, respectively. The 1- and 2-year LC rates were 100% and 88%, respectively. The authors concluded that SBRT may be an effective and safe treatment option in selected patients with BCLM. Mahadevan et al. [44] reported the results after SBRT of a total of 427 patients with liver metastases from different origin including 42 patients with BCLM. At a median follow-up of 14 months (1–91 months) the median OS for patients with BCLM was 21 months. In the whole cohort, smaller tumor volumes (<40 cm^3^) and BED10 ≥ 100 Gy correlated with improved OS ((25 months vs. 15 months, *p* = 0.0014) and (27 months vs. 15 months *p* < 0.0001)), respectively. In BCLM the LC rate after 2 years was 24%.

Hypoxia particularly within large lesions may cause local failure [72] and the distance between treated lesions and the surrounding visceral organs at risk should be more than 8 mm [71]. Liver SBRT is technically challenging, requiring daily imaging guidance and insertion of fiducial markers and/or image fusion to localize the target and assess respiration-related organ motion [44]. The patient selection criteria, and optimal dose and fractionation for liver SBRT are still under investigation. 

#### 3.3.2. Interstitial Brachytherapy (BT)

BT is a type of radiotherapy where a small amount of radioactive material sealed in catheters, wires, needles, or seeds is directly inserted into the tumor tissue. Wieners et al. [45] introduced a technique of interstitial BT applied with CT guidance and 3D CT dataset for exact dose planning. In 41 consecutive patients with 115 BLCM with a median tumor size of 4.6 cm (1.5–11 cm), the CR, PFS, and OS rates at 12 months were 93.5%, 40%, and 79%, respectively. One postinterventional hemorrhage was the only major complication that was encountered. The authors concluded that CT-guided BT is a safe and effective treatment, however further studies are needed to identify best candidates for BT as long-term data is missing.

#### 3.3.3. Thermal Ablation

Thermal ablation methods are minimally invasive, potentially curative, low-risk procedures for local tumor treatment [73,74,75]. In RFA an alternating current is flowing between the uninsulated probe tip and a dispersive skin electrode (unipolar) or between the different electrodes within one or multiple probes (multipolar). Radiofrequency current is converted into tissue heating by friction of the ions in close vicinity to the uninsulated tip of the RFA electrode [76,77,78]. MWA is based on an electromagnetic field (0.9–2.450 GHz), that radiates from an antenna. Water molecules in the surrounding tissue are forced to continuously realign with the oscillating electric field. The kinetic energy rise of the polar water molecules induces heat in the tissue adjacent to the antenna [79]. In contrast to RFA, microwave probes provide faster tissue heating over a larger volume with a less prominent ‘heat sink effect’. With the latest generation microwave antenna, a spherical shaped ablation zone with a short axis diameter of up to 4 cm can be achieved [80]. Cryoablation is based on local tissue destruction based on very low temperatures inducing cellular dehydration, protein denaturation and microcirculatory failure [81]. 

Thermal ablation is considered the first choice for treatment of unresectable liver malignancies. If similar local recurrence rates can be achieved, minimally invasive thermal ablation may serve as an attractive alternative to resection. In contrast to surgical resection with reported overall mortality rates of 5.8 percent in a total of 110,332 liver procedures [16], thermal ablation is associated with a very low complication rate. In a meta-analysis in 9531 patients the reported mortality and major morbidity rates after RFA of liver tumors were 0.15% and 3.29%, respectively [17]. 

Unfortunately, the reported local recurrence rates after conventional CT-/US- guided RFA of BCLM range between 14% and 50% [82,83]. Especially in large lesions the results after thermal ablation are still unacceptable independent of tumor etiology. Therefore, in colorectal liver metastases the sole use of thermal ablation is currently only recommended for liver metastases <3 cm [84]. The size of the ablation zone should cover the entire tumor including a safety margin (0.5–1 cm) of unaffected surrounding tissue [74]. For large lesions, multiple overlapping ablation zones are required [77,85]. Complex planning and placement of multiple probes/electrodes/coaxial needles are difficult to achieve with conventional ultrasound and CT guidance techniques only. Therefore, frameless stereotactic navigation systems in combination with neurosurgical aiming devices [86] are applied for sophisticated 3D planning, translation of the virtual plan into the real patients, and intraoperative confirmation of the ablation margins [87,88] In a recent retrospective study, the efficacy of the so-called stereotactic radiofrequency ablation (SRFA) with intraprocedural image fusion was evaluated for treatment of HCC by histopathological examination of explanted livers in 97 patients, who were treated by SRFA before liver transplantation. Complete pathological response in the explanted liver specimen was achieved in 183 of 188 nodules (97.3%), and in 50 of 52 nodules ≥3 cm (96.2%) [89]. In addition, reported local control and survival rates after SRFA of intrahepatic cholangiocellular carcinoma [18], colorectal liver metastases [13], and melanoma liver metastases [15] were at least comparable to the surgical literature. In all studies tumor size was not related to an increase of local recurrence rate or a decrease of the survival rate.

##### Results after Thermal Ablation of BCLM

Veltri et al. [12] reported the results after ultrasound guided RFA of 45 patients with 87 BCLM. After a mean follow-up of 30 months the local recurrence rate was 19.7%, with a time to local progression of 8 months. Local recurrence rate was significantly influenced by the BCLM diameter. OS at 1 and 3 years was 90% and 44%. Carrafiello et al. [90] treated 13 female patients with 21 BCLM by ultrasound guided RFA. No complications were observed. A mean OS of 10.9 months after RFA was achieved. Lawes et al. [14] evaluated the effectiveness of RFA as a cytoreductive strategy in the management of BCLM in 19 patients including 11 patients with additional stable extrahepatic disease. After a median follow-up of 15 months, 13 patients were alive, with a survival rate of 41.6% at 30 months. 

Bai et al. [20] treated 69 patients with 135 BCLM with ultrasound-guided percutaneous RFA. Major complications occurred in one of the 92 sessions (1.1%). The authors reported a local tumor progression in 11.6% (8/69) of patients, a median OS of 26 months, and the 1-, 3-, and 5 -year survival rates of 81.8%, 25.3% and 11.0%, respectively.

Jakobs et al. [91] treated 111 BCLM in 43 patients with conventional percutaneous CT-guided RFA and achieved a local recurrence rate of 13.5% and a median OS of 58.6 months and a median time to progression of 10.5 months from the date of RFA. Hormone receptor status, HER2 overexpression, and presence of isolated bone metastases did not significantly influence survival. However, extrahepatic disease with the exception of skeletal metastases was associated with a shorter survival time. In a similar study in 12 patients Sofocleous et al. [83] reported a median OS of 60 months after a median follow-up of 22.5 months, with 3- and 5-year OS rates after RFA of 70% and 30%, respectively. The median primary local progression-free interval was 12 months.

Meloni et al. [82] treated 52 patients with BCLM with percutaneous US-guided RFA and reported a median OS of 29.9 months and a 5-year OS rate of 27%. Local tumor progression was observed in 25% (13 of 51) of patients. New intrahepatic metastases occurred in 53% of patients. Patients with large tumors (>2.5 cm in diameter) had a worse prognosis as compared to patients with smaller tumors (hazard ratio: 2.1). 

Kuemler et al. [92] reported a local recurrence rate of 22% after percutaneous US-guided RFA of BCLM in 32 consecutive patients. The median time to intrahepatic progression was 11 months (range 1.6–184 months) and the median survival after first RFA was 33.5 months, with an OS of 87% and 48%, at 1 and 3 years, respectively. 

Positive prognostic factors for survival after RFA were absence of extrahepatic disease [20,91], small BCLM (<2.5 cm) [20,82], complete response after ablation [20] and positive hormone receptor status [20].

Zhang et al. [19] treated 17 patients with 39 BCLM with a median tumor size of 3.5 cm (range: 2–5 cm) by cryoablation. They reported no major complications, a LR rate of 15.4% and a 1-year OS of 70.6%. The authors concluded that cryoablation is a safe and effective treatment, however further studies are needed as long-term data is missing.

Our group [93] reported initial experiences with stereotactic radiofrequency ablation (SRFA) for the treatment of 64 drug resistant BCLM in 26 patients. Despite the inclusion of lesions up to 8.5 cm, a complete local response was achieved in 59/64 (92.2%) of the tumors, with no significant differences (*p* = 0.662) when comparing tumor sizes <3 cm, 3–5 cm and >5 cm. This local control rate is well comparable to the reported R0 rates after surgical resection, which range from 62 to 96%. Estimated median OS and DFS from SRFA treatment were 29.3 and 32 months after a median follow-up of 23 months. In contrast to other studies using conventional image guidance no significant differences (*p* = 0.891) in survival were observed when comparing tumor sizes <3 cm (48.1 ± 13.5 months, median 15.0) vs. 3–5 cm (37.4 ± 5.7 months, median 51.1) vs. >5 cm (21.2 ± 4.8 months, median 20.9). As described above, the selection of the ideal patients is key to achieve long-term survival. In this group 31% of the patients suffered from extrahepatic disease and 83% from multiple BCLM, respectively. 

Reported survival rates for percutaneous RFA in selected patients with BCLM confined to the liver or with stable extrahepatic metastases are comparable to those obtained with resection. Conventional CT- and US-guided RFA and MWA should be used for small lesions and stereotactic RFA and MWA for large lesions. RFA is a safe technique that can be repeated in the case new BCLM appear. When compared with surgical resection, thermal ablation is less invasive, less expensive, has fewer contraindications. and is easier to repeat in case of disease recurrence. Since many patients will develop BCLM after surgical resection, application of the test-of-time approach [91] by applying thermal ablation as initial treatment may avoid unnecessary surgical resections in patients who would develop new metastases. Despite the lack of randomized studies minimally invasive RFA/SRFA may be considered as first line treatment in selected patients with BCLM confined to the liver or with stable extrahepatic disease. 

## 4. Conclusions

In a selected group of patients with oligometastatic disease, effective local treatment of BCLM achieves a survival advantage over systemic chemotherapy and/or hormonal therapy alone. To maximize survival and minimize unnecessary operative morbidity, multiple criteria reflecting the biology of the disease including response to systemic therapy, hormone receptor status, and extent of the disease have to be carefully considered for the determination of appropriate candidates and the ideal timing for local treatment. Further studies are required to better identify those subgroups of patients for whom a multidisciplinary treatment approach with curative intention might be an option. 

Transarterial locoregional treatments including TACE and SIRT may be applied in selected patients with chemo-resistant advanced metastatic liver disease. 

Percutaneous thermal ablation methods, such as conventional CT- and US-guided RFA and MWA for small lesions, and stereotactic RFA and MWA for large lesions, seem to be an attractive alternative to surgical resection. They enable a tissue sparing and cost-saving local curative treatment approach paired with a low complication rate. The decision for local curative treatment of BCLM might therefore be easier if treatment options with similar potential for local control but lower morbidity and mortality as compared to surgical resection are available. In addition, ablation procedures allow to access tumors that are surgically not treatable due to their location or patient comorbidities. 

## Figures and Tables

**Table 1:** Studies included for systematic review showing key study parameters.

Study	Tr.	No. P.	No. T. *n*/$\bar{x}$/%s	Size_†‡_	EHM%	CR/R0%	FU_†_	OS $\tilde{x}$/3Y/5Y	DFS_†_	Positive Prognostic Factors
Bai et al. [12]	RFA	69	135/2/51	2.9 (1–6)	46	88	26	26/25/11	24	Small tumor size, positive hormone receptor status, margin size, no EHD
Carrafiello et al. [13]	RFA	13	21/1.6/62	3.5 (0.5–7)_†_	46	67	12.9	10.9/NR/NR	NR	NR
Jakobs et al. [12]	RFA	43	111/2.6/NR	2.1 (0.5–8.5)_‡_	42	86	37	58.6/NR/NR	10.5	No EHD
Kumler et al. [14]	RFA	32	NR/2/26	2 (0.9–5)_†_	47	78	NR	33.5/48/NR	11	NR
Lawes et al. [15]	RFA	19	46/2.4/58	3_‡_	58	63	15	NR/NR/NR	NR	NR
Meloni et al. [16]	RFA	52	87/1.7/NR	2.5 (0.7–5)_‡_	52	25	19	29.9/43/27	NR	BCLM < 2.5 cm
Sofocleus et al. [17]	RFA	12	14/1.2/86	NR	83	5	22.5	60/70/30	12	NR
Veltri et al. [18]	RFA	45	87/1.9/6	2.3 (1–4.5)_†_	40	74	30	NR/44/NR	8	NR
Zhang et al. [19]	CRA	17	39/2/18	3.5 (2–5)_†_	NR	8785	NR	NR/NR/NR	NR	NR
Bale et al. [20]	SRFA	26	64/2.5/17	2 (0.4–0.5)_†_ 14% > 5	31	92	23	29.3/NR/NR	31.6	NR
Abbott et al. [21]	HR	86	NR/NR/62	15% > 5_†_	28	NR	62	57/NR/NR	14.2	Positive hormone receptor status, preoperative stable disease
Adam et al. [3]	HR	85	NR/NR/37	2.8 (1–19)_†_	32	65	38	32/NR/37	12	Response to preoperative chemotherapy, R0/R1 resection
Bacalbasa et al. [22]	HR	67	NR/NR/49	NR	NR	93	NR	NR/94/69	NR	Positive hormone receptor status
Dittmar et al. [23]	HR	34	50/1.5/35	4 (0–13)_†_	18	62	NR	36/NR/28	NR	HER2 expression, no EHD, age <50 years
Caralt et al. [24]	HR	12	NR/NR/NR	NR	8	83	36	36/79/33	NR	NR
Carlini et al. [25]	HR	17	NR/NR/88	NR	0	NR	NR	NR/NR/46	53	NR
Elias et al. [26]	HR	42	209/5/18	3.2 (0.4–11.1)_†_	17	82	32	34/50/34	16	Positive hormone receptor status
Ercolani et a. [27]	HR	51	NR/NR/47	4 (1–11)_†_	0		61	51/69/36	41	Small tumor diameter, Positive hormone receptor status, triple negative status
He et al. [28]	HR	67	NR/NR/64	4.2 ± 2.2_‡_	21	96	NR	NR/74/32	NR	>2 years between primary and BCLM
Hoffman et al. [29]	HR	41	NR/2/49	15% > 5	29	78	34	58/75/31	34	R0/R1, Late onset of BCLM
Kostov et al. [30]	HR	42	NR/NR/52	5.1 (1.4–9)_†_	48	83	60	43/64/38	29	BCLM size <4 cm, R0, negative portal LN, Response to CTX, positive hormone receptor status
Lubrano J et al. [31]	HR	16	0/0/75	3.5 (1–10)_†_	0		28	42/61/33	NR	Negative hormone receptor status, low number of metastases, minor surgery, age >50, isolated BCLM
Margonis et al. [32]	HR	131	NR/1/NR	3 (2–5)_†_	13	91	24	53/75/NR	24	Negative margin (R0), small diameter of the liver metastasis
Mariani et al. [33]	HR	100	NR/NR/65	1.8 (0.5–11)_†_	7	86	NR	NR/73/5	NR	
Martinez et al. [34]	HR	20	NR/NR/NR	NR	NR		39	32/61/33	NR	Anatomic resections, positive hormone receptor status, age >50 years
Ruiz et al. [10]	HR	139	322/2.3/41	1.8	0	NR	69	73/78/57	NR	NR
Selzner et al. [35]	HR	17	22/1.3/71	2.5 (1.5–5)_†_	18		17	24/NR/22	NR	Late onset of BCLM
van Walsum et al. [36]	HR	32	NR/NR/69	2.5 (0.5–9)_†_	16	69	26	55/NR/37	11	Solitary BCLM
Pocard 2001 et al. [37]	HR	52	NR/NR/69	3.8 (0.4–12)_†_	23	86	23	42/49/NR	NR	Late onset of BCLM, low N stage
Sabol et al. [38]	HR	15	31/2/6	2.2 (0.2–6.6)_†_	33	1	NR	53/67/38	NR	NR
Sakamoto et al. [39]	HR	34	NR/NR/0	4 (1.3–8)_†_	26	NR	72	36/52/21	NR	No EHD
Weinreich et al. [40]	HR	21	NR/NR/55	NR	0	NR	22	53/83/33	NR	R0 resection, low T- and N-stages as well as a low-grade histopathology of the primary tumor
Vertriest et al. [41]	HR	27	38/1.4/56	3.9 ± 2.3_‡_	4	89	52	116/83/78	NR	Stage of primary tumor, Solitary lesions
Yoshimoto et al. [42]	HR	25	NR/NR/56	4.1 (1.3–7)_†_	32	NR	NR	34/NR/27	24	NR
Onal et al. [43]	SBRT	22	29/1.3/86	2.1_‡_	32	88	16	NR/NR/NR	7.4	NR
Mahadevan et al. [44]	SBRT	42	NR/NR/NR	NR	NR	NR	14	22/14/5	NR	BCLM < 40 cm^3^; BED10 ≥ 100 Gy
Wieners et al. [45]	BT	41	115/NR/	4.6 (1.5–11)_†_	NR	94	18	NR/NR/NR	NR	Extent of pre-treatment
Cianni et al. [46]	SIRT	52	NR/NR/0	NR	46	0	NR	11.5/NR/NR	NR	NR
Fendler et al. [47]	SIRT	81	NR/NR/0	NR	67	0	NR	8.7/0/0	NR	NR
Gordon et al. [48]	SIRT	75	NR/NR/15	NR	77	NR	NR	6.6/NR/NR	3.2	Solitary BCLM, Tumor burden
Haug et al. [49]	SIRT	58	NR/NR/NR	NR	66	NR	2.3	4/NR/NR	NR	Responder
Jakobs et al. [50]	SIRT	30	NR/NR/0	NR	57	0	14	11.7/NR/NR	NR	No EHD
Pieper et al. [51]	SIRT	44	NR/NR/2	NR	89	0	4	6.1/0/0	3.4 TTP	ECOG status <1, small liver tumor burden, No EHD, response, vascularity
Saxena et al. [52]	SIRT	40	NR/NR/0	NR	6	5	11.2	13.6/0/0	6.8 TTP	Low tumor burden, CTX after SIRT, response
Eichler et al. [53]	TACE	43	NR/NR/NR	NR	49	02	4	10.2/NR/NR	3.3	Low vascularized tumors
Li et al. [54]	TACE	28	NR/NR/32	2.8 (1–8)_†_	40	07	28	28/13/NR	NR	N status of the primary tumor, clinical stage of BCLM, Child–Pugh grade

Tr. = Local treatment, No. P. = number of patients, No. T. T/$\bar{x}$/%s = number of tumors, total number/mean/% solitary, EHD = extrahepatic disease, BCLM = breast cancer liver metastases, CR = complete response, $\tilde{x}$, † = median, $\bar{x}$, ‡ = mean, FU = follow-up, NR = not reported, HR = hepatic resection, RFA = radiofrequency ablation, SRFA = stereotactic radiofrequency ablation, SBRT = stereotactic body radiation therapy, TACE = transarterial chemoembolization, BT = brachytherapy, CRA = cryoablation, SIRT = selective internal brachytherapy, BED = radiation biologically effective dose, ECOG = eastern cooperative oncology group, CTX = chemotherapy.

**Table 2:** Summarized key features according to treatment option.

Treatment Option	No. of included Studies	No. P.	Size_†‡_	EHM%	CR/R0%	OS†	DFS_†_	Strength /Weakness
RFA	8	203	2–2.5 (0.5–5)	40–83	5–88	11–60	8–24	low morbidity, repeatability/insufficient local control in large tumors
CRA	1	17	3.5 (2–5)	NR	85	NR	NR	low morbidity/no long-term data, single center
SRFA	1	26	2 (0.4–8.5)_†_	46	92	29.3	31.6	low morbidity, good local tumor control in small and large tumors/single center
HR	24	1173	1.8–5.1 (0.4–19)	0–48	62–96	24–116	11–53	good local tumor control in small and large tumors/high morbidity, limited repeatability
SBRT	2	64	2.1/NR	32	88	22/NR	7.4/NR	low morbidity/high recurrence, short survival time
BT	1	41	4.6 (1.5–11)	NR	93.5	NR	NR	low morbidity/no long-term data, single center
SIRT	7	380	NR	6–89	0–5	4–14	3.2/NR	low morbidity/palliative
TACE	2	71	2.8 (1–8)/NR	40–49	2–7	10–28	3.3/NR	low morbidity/ palliative

Tr. = local treatment, No. P. = number of patients, No. T. T/$\bar{x}$/%s = number of tumors, total number/mean/% solitary, EHM = extrahepatic metastases, $\tilde{x}$, † = median, $\bar{x}$, ‡ = mean, FU = follow-up, HR = hepatic resection, RFA = radiofrequency ablation, SRFA = stereotactic radiofrequency ablation, SBRT = stereotactic body radiation therapy, TACE = transarterial chemoembolization, BT = brachytherapy, CRA = cryoablation.

## References

[1] Siegel R.L., Miller K.D., Jemal A. (2018). Cancer statistics, 2018. CA Cancer J. Clin..

[2] He Z.Y., Wu S.G., Peng F., Zhang Q., Luo Y., Chen M., Bao Y. (2017). Up-Regulation of RFC3 Promotes Triple Negative Breast Cancer Metastasis and is Associated with Poor Prognosis Via EMT. Transl. Oncol..

[3] Adam R., Aloia T., Krissat J., Bralet M.P., Paule B., Giacchetti S., Delvart V., Azoulay D., Bismuth H., Castaing D. (2006). Is liver resection justified for patients with hepatic metastases from breast cancer?. Ann. Surg..

[4] Leung A.M., Vu H.N., Nguyen K.A., Thacker L.R., Bear H.D. (2010). Effects of surgical excision on survival of patients with stage IV breast cancer. J. Surg. Res..

[5] Pivot X., Asmar L., Hortobagyi G.N., Theriault R., Pastorini F., Buzdar A. (2000). A retrospective study of first indicators of breast cancer recurrence. Oncology.

[6] Eng L.G., Dawood S., Sopik V., Haaland B., Tan P.S., Bhoo-Pathy N., Warner E., Iqbal J., Narod S.A., Dent R. (2016). Ten-year survival in women with primary stage IV breast cancer. Breast Cancer Res. Treat..

[7] Cristofanilli M., Hortobagyi G.N. (2001). New horizons in treating metastatic disease. Clin. Breast Cancer.

[8] Weichselbaum R.R., Hellman S. (2011). Oligometastases revisited. Nat. Rev. Clin. Oncol..

[9] Ruiz A., Castro-Benitez C., Sebagh M., Giacchetti S., Castro-Santa E., Wicherts D.A., van Hillegersberg R., Paule B., Castaing D., Morère J.-F. (2015). Repeat Hepatectomy for Breast Cancer Liver Metastases. Ann. Surg. Oncol..

[10] Ruiz A., van Hillegersberg R., Siesling S., Castro-Benitez C., Sebagh M., Wicherts D.A., de Ligt K.M., Goense L., Giacchetti S., Castaing D. (2018). Surgical resection versus systemic therapy for breast cancer liver metastases: Results of a European case matched comparison. Eur. J. Cancer.

[11] Cardoso F., Costa A., Senkus E., Aapro M., Andre F., Barrios C.H., Bergh J., Bhattacharyya G., Biganzoli L., Cardoso M.J. (2017). 3rd ESO-ESMO International Consensus Guidelines for Advanced Breast Cancer (ABC 3). Ann. Oncol..

[12] Veltri A., Gazzera C., Barrera M., Busso M., Solitro F., Filippini C., Garetto I. (2014). Radiofrequency thermal ablation (RFA) of hepatic metastases (METS) from breast cancer (BC): An adjunctive tool in the multimodal treatment of advanced disease. Radiol. Med..

[13] Bale R., Widmann G., Schullian P., Haidu M., Pall G., Klaus A., Weiss H., Biebl M., Margreiter R. (2012). Percutaneous stereotactic radiofrequency ablation of colorectal liver metastases. Eur. Radiol..

[14] Lawes D., Chopada A., Gillams A., Lees W., Taylor I. (2006). Radiofrequency ablation (RFA) as a cytoreductive strategy for hepatic metastasis from breast cancer. Ann. R. Coll. Surg. Engl..

[15] Bale R., Schullian P., Schmuth M., Widmann G., Jaschke W., Weinlich G. (2016). Stereotactic Radiofrequency Ablation for Metastatic Melanoma to the Liver. Cardiovasc Intervent Radiol..

[16] Filmann N., Walter D., Schadde E., Bruns C., Keck T., Lang H., Oldhafer K., Schlitt H.J., Schön M.R., Herrmann E. (2019). Mortality after liver surgery in Germany. Br. J. Surg..

[17] Bertot L.C., Sato M., Tateishi R., Yoshida H., Koike K. (2011). Mortality and complication rates of percutaneous ablative techniques for the treatment of liver tumors: A systematic review. Eur. Radiol..

[18] Haidu M., Dobrozemsky G., Schullian P., Widmann G., Klaus A., Weiss H., Margreiter R., Bale R. (2012). Stereotactic radiofrequency ablation of unresectable intrahepatic cholangiocarcinomas: A retrospective study. Cardiovasc Intervent Radiol..

[19] Zhang W., Yu H., Guo Z., Li B., Si T., Yang X., Wang H. (2014). Percutaneous cryoablation of liver metastases from breast cancer: Initial experience in 17 patients. Clin. Radiol..

[20] Bai X.M., Yang W., Zhang Z.Y., Jiang A.N., Wu W., Lee J.C., Chen M.H., Yan K. (2019). Long-term outcomes and prognostic analysis of percutaneous radiofrequency ablation in liver metastasis from breast cancer. Int J. Hyperthermia..

[21] Abbott D.E., Brouquet A., Mittendorf E.A., Andreou A., Meric-Bernstam F., Valero V., Green M.C., Kuerer H.M., Curley S.A., Abdalla E.K. (2012). Resection of liver metastases from breast cancer: Estrogen receptor status and response to chemotherapy before metastasectomy define outcome. Surgery.

[22] Bacalbasa N., Balescu I., Ilie V., Florea R., Sorop A., Brasoveanu V., Brezean I., Vilcu M., Dima S., Popescu I. (2018). The Impact on the Long-term Outcomes of Hormonal Status After Hepatic Resection for Breast Cancer Liver Metastases. In Vivo.

[23] Dittmar Y., Altendorf-Hofmann A., Schule S., Ardelt M., Dirsch O., Runnebaum I.B., Settmacher U. (2013). Liver resection in selected patients with metastatic breast cancer: A single-centre analysis and review of literature. J. Cancer Res. Clin. Oncol..

[24] Caralt M., Bilbao I., Cortes J., Escartin A., Lazaro J.L., Dopazo C., Olsina J.J., Balsells J., Charco R. (2008). Hepatic resection for liver metastases as part of the “oncosurgical” treatment of metastatic breast cancer. Ann. Surg. Oncol..

[25] Carlini M., Lonardo M.T., Carboni F., Petric M., Vitucci C., Santoro R., Lepiane P., Ettorre G.M., Santoro E. (2002). Liver metastases from breast cancer. Results of surgical resection. Hepatogastroenterology.

[26] Elias D., Maisonnette F., Druet-Cabanac M., Ouellet J.-F., Guinebretiere J.-M., Spielmann M., Delaloge S. (2003). An attempt to clarify indications for hepatectomy for liver metastases from breast cancer. Am. J. Surg..

[27] Ercolani G., Zanello M., Serenari M., Cescon M., Cucchetti A., Ravaioli M., Del Gaudio M., D’Errico A., Brandi G., Pinna A.D. (2018). Ten-Year Survival after Liver Resection for Breast Metastases: A Single-Center Experience. Dig. Surg..

[28] He X., Zhang Q., Feng Y., Li Z., Pan Q., Zhao Y., Zhu W., Zhang N., Zhou J., Wang L. (2019). Resection of liver metastases from breast cancer: A multicentre analysis. Clin. Transl. Oncol..

[29] Hoffmann K., Franz C., Hinz U., Schirmacher P., Herfarth C., Eichbaum M., Buchler M.W., Schemmer P. (2010). Liver resection for multimodal treatment of breast cancer metastases: Identification of prognostic factors. Ann. Surg. Oncol..

[30] Kostov D.V., Kobakov G.L., Yankov D.V. (2013). Prognostic factors related to surgical outcome of liver metastases of breast cancer. J. Breast Cancer.

[31] Lubrano J., Roman H., Tarrab S., Resch B., Marpeau L., Scotte M. (2008). Liver resection for breast cancer metastasis: Does it improve survival?. Surg. Today.

[32] Margonis G.A., Buettner S., Sasaki K., Kim Y., Ratti F., Russolillo N., Ferrero A., Berger N., Gamblin T.C., Poultsides G. (2016). The role of liver-directed surgery in patients with hepatic metastasis from primary breast cancer: A multi-institutional analysis. HPB.

[33] Mariani P., Servois V., De Rycke Y., Bennett S.P., Feron J.G., Almubarak M.M., Reyal F., Baranger B., Pierga J.Y., Salmon R.J. (2013). Liver metastases from breast cancer: Surgical resection or not? A case-matched control study in highly selected patients. Eur. J. Surg. Oncol..

[34] Martinez S.R., Young S.E., Giuliano A.E., Bilchik A.J. (2006). The utility of estrogen receptor, progesterone receptor, and Her-2/neu status to predict survival in patients undergoing hepatic resection for breast cancer metastases. Am. J. Surg..

[35] Selzner M., Morse M.A., Vredenburgh J.J., Meyers W.C., Clavien P.A. (2000). Liver metastases from breast cancer: Long-term survival after curative resection. Surgery.

[36] van Walsum G.A., de Ridder J.A., Verhoef C., Bosscha K., van Gulik T.M., Hesselink E.J., Ruers T.J., van den Tol M.P., Nagtegaal I.D., Brouwers M. (2012). Resection of liver metastases in patients with breast cancer: Survival and prognostic factors. Eur. J. Surg. Oncol..

[37] Pocard M., Pouillart P., Asselain B., Salmon R. (2000). Hepatic resection in metastatic breast cancer: Results and prognostic factors. Eur. J. Surg. Oncol..

[38] Sabol M., Donat R., Chvalny P., Dyttert D., Palaj J., Durdik S. (2014). Surgical management of breast cancer liver metastases. Neoplasma.

[39] Sakamoto Y., Yamamoto J., Yoshimoto M., Kasumi F., Kosuge T., Kokudo N., Makuuchi M. (2005). Hepatic resection for metastatic breast cancer: Prognostic analysis of 34 patients. World J. Surg..

[40] Weinrich M., Weiss C., Schuld J., Rau B.M. (2014). Liver resections of isolated liver metastasis in breast cancer: Results and possible prognostic factors. HPB Surg..

[41] Vertriest C., Berardi G., Tomassini F., Vanden Broucke R., Depypere H., Cocquyt V., Denys H., Van Belle S., Troisi R.I. (2015). Resection of single metachronous liver metastases from breast cancer stage I-II yield excellent overall and disease-free survival. Single center experience and review of the literature. Dig. Surg..

[42] Yoshimoto M., Tada T., Saito M., Takahashi K., Uchida Y., Kasumi F. (2000). Surgical treatment of hepatic metastases from breast cancer. Breast Cancer Res. Treat..

[43] Onal C., Guler O.C., Yildirim B.A. (2018). Treatment outcomes of breast cancer liver metastasis treated with stereotactic body radiotherapy. Breast.

[44] Mahadevan A., Blanck O., Lanciano R., Peddada A., Sundararaman S., D’Ambrosio D., Sharma S., Perry D., Kolker J., Davis J. (2018). Stereotactic Body Radiotherapy (SBRT) for liver metastasis-clinical outcomes from the international multi-institutional RSSearch(R) Patient Registry. Radiat. Oncol..

[45] Wieners G., Mohnike K., Peters N., Bischoff J., Kleine-Tebbe A., Seidensticker R., Seidensticker M., Gademann G., Wust P., Pech M. (2011). Treatment of hepatic metastases of breast cancer with CT-guided interstitial brachytherapy-a phase II-study. Radiother. Oncol..

[46] Cianni R., Pelle G., Notarianni E., Saltarelli A., Rabuffi P., Bagni O., Filippi L., Cortesi E. (2013). Radioembolisation with (90)Y-labelled resin microspheres in the treatment of liver metastasis from breast cancer. Eur. Radiol..

[47] Fendler W.P., Lechner H., Todica A., Paprottka K.J., Paprottka P.M., Jakobs T.F., Michl M., Bartenstein P., Lehner S., Haug A.R. (2016). Safety, Efficacy, and Prognostic Factors After Radioembolization of Hepatic Metastases from Breast Cancer: A Large Single-Center Experience in 81 Patients. J. Nucl. Med..

[48] Gordon A.C., Gradishar W.J., Kaklamani V.G., Thuluvath A.J., Ryu R.K., Sato K.T., Gates V.L., Salem R., Lewandowski R.J. (2014). Yttrium-90 radioembolization stops progression of targeted breast cancer liver metastases after failed chemotherapy. J. Vasc. Interv. Radiol..

[49] Haug A.R., Tiega Donfack B.P., Trumm C., Zech C.J., Michl M., Laubender R.P., Uebleis C., Bartenstein P., Heinemann V., Hacker M. (2012). 18F-FDG PET/CT predicts survival after radioembolization of hepatic metastases from breast cancer. J. Nucl. Med..

[50] Jakobs T.F., Hoffmann R.T., Fischer T., Stemmler H.J., Tatsch K., La Fougere C., Murthy R., Reiser M.F., Helmberger T.K. (2008). Radioembolization in patients with hepatic metastases from breast cancer. J. Vasc. Interv. Radiol..

[51] Pieper C.C., Meyer C., Wilhelm K.E., Block W., Nadal J., Ahmadzadehfar H., Willinek W.A., Schild H.H. (2016). Yttrium-90 Radioembolization of Advanced, Unresectable Breast Cancer Liver Metastases-A Single-Center Experience. J. Vasc. Interv. Radiol..

[52] Saxena A., Kapoor J., Meteling B., Morris D.L., Bester L. (2014). Yttrium-90 radioembolization for unresectable, chemoresistant breast cancer liver metastases: A large single-center experience of 40 patients. Ann. Surg. Oncol..

[53] Eichler K., Jakobi S., Gruber-Rouh T., Hammerstingl R., Vogl T.J., Zangos S. (2013). Transarterial chemoembolisation (TACE) with gemcitabine: Phase II study in patients with liver metastases of breast cancer. Eur. J. Radiol..

[54] Li X.-P., Meng Z.-Q., Guo W.-J., Li J. (2005). Treatment for liver metastases from breast cancer: Results and prognostic factors. World J. Gastroenterol..

[55] Fairhurst K., Leopardi L., Satyadas T., Maddern G. (2016). The safety and effectiveness of liver resection for breast cancer liver metastases: A systematic review. Breast.

[56] Bergenfeldt M., Jensen B.V., Skjoldbye B., Nielsen D. (2011). Liver resection and local ablation of breast cancer liver metastases--a systematic review. Eur. J. Surg. Oncol..

[57] Lermite E., Marzano E., Chereau E., Rouzier R., Pessaux P. (2010). Surgical resection of liver metastases from breast cancer. Surg. Oncol..

[58] Vlastos G., Smith D.L., Singletary S.E., Mirza N.Q., Tuttle T.M., Popat R.J., Curley S.A., Ellis L.M., Roh M.S., Vauthey J.N. (2004). Long-term survival after an aggressive surgical approach in patients with breast cancer hepatic metastases. Ann. Surg. Oncol..

[59] Howlader M., Heaton N., Rela M. (2011). Resection of liver metastases from breast cancer: Towards a management guideline. Int. J. Surg..

[60] Elias D., Lasser P., Ducreux M., Duvillard P., Ouellet J.F., Dromain C., Schlumberger M., Pocard M., Boige V., Miquel C. (2003). Liver resection (and associated extrahepatic resections) for metastatic well-differentiated endocrine tumors: A 15-year single center prospective study. Surgery.

[61] Kokudo N., Imamura H., Sugawara Y., Sakamoto Y., Yamamoto J., Seki M., Makuuchi M. (2004). Surgery for multiple hepatic colorectal metastases. J. Hepatobiliary Pancreat. Surg..

[62] Cho S.W., Kitisin K., Buck D., Steel J., Brufsky A., Gillespie R., Tsung A., Marsh J.W., Geller D.A., Gamblin T.C. (2010). Transcatheter arterial chemoembolization is a feasible palliative locoregional therapy for breast cancer liver metastases. Int. J. Surg. Oncol..

[63] Wang M., Zhang J., Ji S., Shao G., Zhao K., Wang Z., Wu A. (2017). Transarterial chemoembolisation for breast cancer with liver metastasis: A systematic review. Breast.

[64] Roche A., Girish B.V., de Baere T., Baudin E., Boige V., Elias D., Lasser P., Schlumberger M., Ducreux M. (2003). Trans-catheter arterial chemoembolization as first-line treatment for hepatic metastases from endocrine tumors. Eur. Radiol..

[65] Llovet J.M., Real M.I., Montaña X., Planas R., Coll S., Aponte J., Ayuso C., Sala M., Muchart J., Solà R. (2002). Arterial embolisation or chemoembolisation versus symptomatic treatment in patients with unresectable hepatocellular carcinoma: A randomised controlled trial. Lancet.

[66] Wasan H.S., Gibbs P., Sharma N.K., Taieb J., Heinemann V., Ricke J., Peeters M., Findlay M., Weaver A., Mills J. (2017). First-line selective internal radiotherapy plus chemotherapy versus chemotherapy alone in patients with liver metastases from colorectal cancer (FOXFIRE, SIRFLOX, and FOXFIRE-Global): A combined analysis of three multicentre, randomised, phase 3 trials. Lancet Oncol..

[67] Riaz A., Lewandowski R.J., Kulik L.M., Mulcahy M.F., Sato K.T., Ryu R.K., Omary R.A., Salem R. (2009). Complications following radioembolization with yttrium-90 microspheres: A comprehensive literature review. J. Vasc. Interv. Radiol..

[68] Gil-Alzugaray B., Chopitea A., Inarrairaegui M., Bilbao J.I., Rodriguez-Fraile M., Rodriguez J., Benito A., Dominguez I., D’Avola D., Herrero J.I. (2013). Prognostic factors and prevention of radioembolization-induced liver disease. Hepatology.

[69] Smits M.L., Prince J.F., Rosenbaum C.E., van den Hoven A.F., Nijsen J.F., Zonnenberg B.A., Seinstra B.A., Lam M.G., van den Bosch M.A. (2013). Intra-arterial radioembolization of breast cancer liver metastases: A structured review. Eur. J. Pharmacol..

[70] Lee M.T., Kim J.J., Dinniwell R., Brierley J., Lockwood G., Wong R., Cummings B., Ringash J., Tse R.V., Knox J.J. (2009). Phase I study of individualized stereotactic body radiotherapy of liver metastases. J. Clin. Oncol..

[71] Scorsetti M., Clerici E., Comito T. (2014). Stereotactic body radiation therapy for liver metastases. J. Gastrointest. Oncol..

[72] Brown J.M., Diehn M., Loo B.W. (2010). Stereotactic ablative radiotherapy should be combined with a hypoxic cell radiosensitizer. Int. J. Radiat. Oncol. Biol. Phys..

[73] Livraghi T., Goldberg S.N., Lazzaroni S., Meloni F., Ierace T., Solbiati L., Gazelle G.S. (2000). Hepatocellular carcinoma: Radio-frequency ablation of medium and large lesions. Radiology.

[74] Solbiati L., Livraghi T., Goldberg S.N., Ierace T., Meloni F., Dellanoce M., Cova L., Halpern E.F., Gazelle G.S. (2001). Percutaneous radio-frequency ablation of hepatic metastases from colorectal cancer: Long-term results in 117 patients. Radiology.

[75] Mulier S., Ni Y., Jamart J., Ruers T., Marchal G., Michel L. (2005). Local recurrence after hepatic radiofrequency coagulation. Ann. Surg..

[76] Goldberg S.N., Hahn P.F., Tanabe K.K., Mueller P.R., Schima W., Athanasoulis C.A., Compton C.C., Solbiati L., Gazelle G.S. (1998). Percutaneous radiofrequency tissue ablation: Does perfusion-mediated tissue cooling limit coagulation necrosis?. J. Vasc. Interv. Radiol..

[77] Chen M.H., Wei Y., Yan K., Gao W., Dai Y., Huo L., Yin S.S., Zhang H., Poon R.T. (2006). Treatment strategy to optimize radiofrequency ablation for liver malignancies. J. Vasc. Interv. Radiol..

[78] Dupuy D.E., Goldberg S.N. (2001). Image-guided radiofrequency tumor ablation: Challenges and opportunities--part II. J. Vasc. Interv. Radiol..

[79] Lubner M.G., Brace C.L., Hinshaw J.L., Lee F.T. (2010). Microwave tumor ablation: Mechanism of action, clinical results, and devices. J. Vasc. Interv. Radiol..

[80] Berber E. (2015). The first clinical application of planning software for laparoscopic microwave thermosphere ablation of malignant liver tumours. HPB.

[81] Gage A.A. (1998). History of cryosurgery. Semin. Surg. Oncol..

[82] Meloni M.F., Andreano A., Laeseke P.F., Livraghi T., Sironi S., Lee F.T. (2009). Breast cancer liver metastases: US-guided percutaneous radiofrequency ablation--intermediate and long-term survival rates. Radiology.

[83] Sofocleous C.T., Nascimento R.G., Gonen M., Theodoulou M., Covey A.M., Brody L.A., Solomon S.M., Thornton R., Fong Y., Getrajdman G.I. (2007). Radiofrequency ablation in the management of liver metastases from breast cancer. AJR Am. J. Roentgenol..

[84] Gillams A., Goldberg N., Ahmed M., Bale R., Breen D., Callstrom M., Chen M.H., Choi B.I., de Baere T., Dupuy D. (2015). Thermal ablation of colorectal liver metastases: A position paper by an international panel of ablation experts, the interventional oncology sans frontieres meeting 2013. Eur. Radiol..

[85] Lee J.M., Han J.K., Kim H.C., Kim S.H., Kim K.W., Joo S.M., Choi B.I. (2007). Multiple-electrode radiofrequency ablation of in vivo porcine liver: Comparative studies of consecutive monopolar, switching monopolar versus multipolar modes. Invest. Radiol..

[86] Bale R.J., Laimer I., Martin A., Schlager A., Mayr C., Rieger M., Czermak B.V., Kovacs P., Widmann G. (2006). Frameless stereotactic cannulation of the foramen ovale for ablative treatment of trigeminal neuralgia. Neurosurgery.

[87] Widmann G., Schullian P., Haidu M., Bale R. (2012). Stereotactic radiofrequency ablation (SRFA) of liver lesions: Technique effectiveness, safety, and interoperator performance. Cardiovasc. Intervent. Radiol..

[88] Bale R., Widmann G., Stoffner D.I. (2010). Stereotaxy: Breaking the limits of current radiofrequency ablation techniques. Eur. Radiol..

[89] Bale R., Schullian P., Eberle G., Putzer D., Zoller H., Schneeberger S., Manzl C., Moser P., Oberhuber G. (2018). Stereotactic Radiofrequency Ablation of Hepatocellular Carcinoma: A Histopathological Study in Explanted Livers. Hepatology.

[90] Carrafiello G., Fontana F., Cotta E., Petulla M., Brunese L., Mangini M., Fugazzola C. (2011). Ultrasound-guided thermal radiofrequency ablation (RFA) as an adjunct to systemic chemotherapy for breast cancer liver metastases. Radiol. Med..

[91] Jakobs T.F., Hoffmann R.T., Schrader A., Stemmler H.J., Trumm C., Lubienski A., Murthy R., Helmberger T.K., Reiser M.F. (2009). CT-guided radiofrequency ablation in patients with hepatic metastases from breast cancer. Cardiovasc. Intervent. Radiol..

[92] Kumler I., Parner V.K., Tuxen M.K., Skjoldbye B., Bergenfeldt M., Nelausen K.M., Nielsen D.L. (2015). Clinical outcome of percutaneous RF-ablation of non-operable patients with liver metastasis from breast cancer. La Radiol. Med..

[93] Bale R., Richter M., Dunser M., Levy E., Buchberger W., Schullian P. (2018). Stereotactic Radiofrequency Ablation for Breast Cancer Liver Metastases. J. Vasc. Interv. Radiol..

